# Pharmacometabolomic Approach to Predict QT Prolongation in Guinea Pigs

**DOI:** 10.1371/journal.pone.0060556

**Published:** 2013-04-04

**Authors:** Jeonghyeon Park, Keumhan Noh, Hae Won Lee, Mi-sun Lim, Sook Jin Seong, Jeong Ju Seo, Eun-Jung Kim, Wonku Kang, Young-Ran Yoon

**Affiliations:** 1 Department of Molecular Medicine, Kyungpook National University School of Medicine and BK21 program, Kyungpook National University School of Medicine, Daegu, South Korea; 2 Clinical Trial Center, Kyungpook National University Hospital, Daegu, South Korea; 3 College of Pharmacy, Yeungnam University, Kyoungbuk, South Korea; 4 National Institute of Food and Drug Safety Evaluation, Korea Food and Drug Administration, Chungbuk, South Korea; Johns Hopkins University School of Medicine, United States of America

## Abstract

Drug-induced torsades de pointes (TdP), a life-threatening arrhythmia associated with prolongation of the QT interval, has been a significant reason for withdrawal of several medicines from the market. Prolongation of the QT interval is considered as the best biomarker for predicting the torsadogenic risk of a new chemical entity. Because of the difficulty assessing the risk for TdP during drug development, we evaluated the metabolic phenotype for predicting QT prolongation induced by sparfloxacin, and elucidated the metabolic pathway related to the QT prolongation. We performed electrocardiography analysis and liquid chromatography–mass spectroscopy-based metabolic profiling of plasma samples obtained from 15 guinea pigs after administration of sparfloxacin at doses of 33.3, 100, and 300 mg/kg. Principal component analysis and partial least squares modelling were conducted to select the metabolites that substantially contributed to the prediction of QT prolongation. QTc increased significantly with increasing dose (r = 0.93). From the PLS analysis, the key metabolites that showed the highest variable importance in the projection values (>1.5) were selected, identified, and used to determine the metabolic network. In particular, cytidine-5′-diphosphate (CDP), deoxycorticosterone, L-aspartic acid and stearic acid were found to be final metabolomic phenotypes for the prediction of QT prolongation. Metabolomic phenotypes for predicting drug-induced QT prolongation of sparfloxacin were developed and can be applied to cardiac toxicity screening of other drugs. In addition, this integrative pharmacometabolomic approach would serve as a good tool for predicting pharmacodynamic or toxicological effects caused by changes in dose.

## Introduction

**Figure 1:**
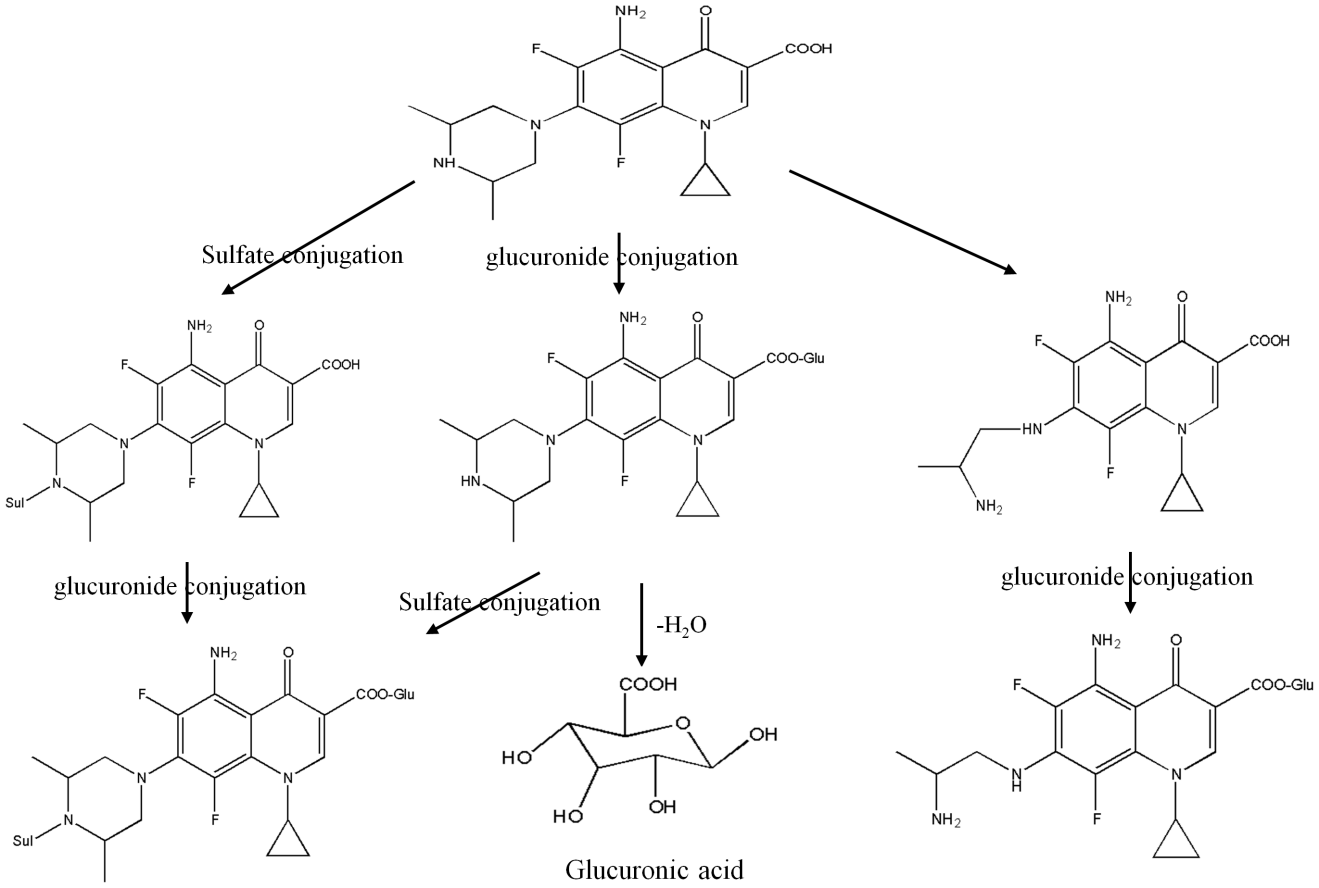
Chemical structure of sparfloxacin and its metabolic pathway.

Currently, a huge antibacterial drug market has developed worldwide, and it is anticipated that side effects caused by the regular administration of such drugs will lead to substantial additional medical expenses [1], [2], [3], [4], [5]. Side effects such as torsades de pointes (TdP) can be damaging to an individual regardless of their rate of incidence; however, TdP can barely be detected using conventional pharmacotoxicological and clinical tests [5], [6], [7]. Because toxic effects may offset the benefits of drug therapy, there has been increasing interest in developing biomarkers that provide an early warning of possible drug toxicity. Because a quantitative relationship can be found between QT interval prolongation and the risk of TdP, this interval is widely used as a biomarker to assess the proarrhythmic risk of drugs [8], [9], [10], [11], [12]. Sparfloxacin (C_19_H_22_F_2_N_4_O_3_; molecular weight, 392.4) is a compound belonging to the third-generation family of fluoroquinolones, with two fluorides in its molecular structure (Figure 1). Sparfloxacin was reported to cause QTc interval prolongation or death from arrhythmia in humans when orally administered [6]. Adamantidis *et al*. demonstrated that sparfloxacin could prolong cardiac repolarisation and induce early afterdepolarisations in rabbit Purkinje fibres [13]. In addition, oral administration of 60 mg/kg sparfloxacin causes TdP, leading to ventricular fibrillation in dogs with chronic complete atrioventricular block [14]. Most of the drugs that induce QT prolongation have been reported to share the same ability to block the rapid component of the delayed rectifier K^+^ current (I_Kr_) encoded by the human ether-à-go-go-related gene (hERG) K^+^ channel [15], [16]. Blockade of I_Kr_ leads to a delay in cardiac repolarisation and prolongs the action potential duration (APD) in myocardia and consequently prolongs the QT interval on electrocardiography (ECG) [17], [18], [19], [20], [21].

Regarding this point, QT interval prolongation has been reported to have I_Kr_-blocking ability [22], [23], [24] and is related to TdP [14]. Thus, determining pharmacometabolomic approaches to evaluate QT prolongation is very important and useful because QT prolongation is one of the most serious cardiovascular toxicities involved in the early stage of drug development.

With the arrival of the post-genomic era, a radical development of genomic and proteomic technologies has occurred. However, the importance of pharmacometabolomic approaches has emerged because of their direct role in the control of body function [25], [26], [27], [28], [29], [30]. Active research is being conducted on the mechanisms of toxic materials and drugs using metabolite analysis [31], [32], [33], [34], [35], [36]. Pharmacometabolomic techniques are also being investigated for their use in interpreting the differences among individuals and among species, as well as among different phenotypes caused by environmental factors [37], [38], [39]. The complete metabolite profile of biological samples such as blood, urine, and tissue provides useful information concerning the diverse physiological or pathological phenomena occurring in the body.

Several pharmacometabolomic studies have provided metabolic profiles that have been useful for investigating drug toxicity. Metabolic profiles of biofluids such as plasma, cerebrospinal fluid, and urine reflect both normal variation in and the pathophysiological impact of toxicity or disease on single or multiple organ systems [25], [28], [29], [30], [31], [33], [34], [35], [36], [37], [40], [41], [42]. Clayton *et al*. (2006) [37] first demonstrated that a predose metabolic profile of urine could predict the toxicity and metabolism of paracetomol in rats. Predose and postdose profiles were obtained again in a similar follow-up study [25]. Predose spectra were modelled in relation to drug metabolite excretion to detect predose biomarkers of drug metabolites. Winnike *et al*. (2010) [35] predicted acetaminophen-induced liver injury from predose human urine samples. Additionally, Nicholson *et al*. (2002) [34] suggested the use of liver and kidney toxicity biomarkers to provide information on drug toxicity.

However, QT prolongation has never been evaluated by pharmacometabolomics. Thus, there is substantial interest in identifying metabolic phenotypes [43] that can predict QT prolongation and thus be used in clinical settings, as well as in understanding the pharmacological roles of such phenotypes. Indeed, metabolic phenotypes have the potential to be most valuable for detecting a predisposition to or risk of drug toxicity, thus increasing the frequency with which these signals can be captured in premarketing clinical trials.

The overall scheme of the proposed method can be summarised as follows. As the first step, large-scale metabolite profiling is conducted, which is essential for the discovery of a metabolic phenotype predictive of drug toxicity [31], [32], [33], [34], [35], [36]. Then, using the biofluid metabolic signatures generated by profiling, principal component analysis (PCA) and partial least squares (PLS) models are used to predict toxicity after drug administration, and the key metabolites that are strongly associated with the toxic variables are selected based on the PLS model. Finally, using the selected key metabolites, a hypothetical network describing the metabolic pathways is constructed.

Here, we present a novel metabolic profile that can predict individualised QT prolongation in guinea pigs administered three different doses (33.3, 100, and 300 mg/kg) of sparfloxacin [44]. A comparative analysis was conducted on endogenous metabolites considering both sparfloxacin and the drug metabolites (Figure 1). We developed an integrative approach that effectively combines liquid chromatography–mass spectroscopy (LC–MS) analysis, PCA, PLS modelling [45], [46], [47], and network analyses. Finally, by integrating the results of the PLS and network analyses, a metabolic phenotype including four metabolites was identified for predicting the QT prolongation of sparfloxacin, which might ultimately be used in clinical practice. This integrative approach can also be extended to other drugs with complex responses and drug toxicity. In addition, numerical formulation of the electrocardiographic changes that are caused by antibacterial quinolones, when established in relation to their pharmacokinetic behaviour after administration, would serve as a good tool for predicting pharmacodynamic or toxicological effects caused by changes in dose.

## Methods

### 1 Study design

#### 1.1 Test drug and materials

Sparfloxacin, sotalol (internal standard), ammonium acetate, and sodium hydroxide were purchased from Sigma (Seoul, Korea), and acetonitrile was obtained from J.T. Baker (Seoul, Korea). All other chemicals and solvents were of high-performance liquid chromatography (HPLC) grade or the highest quality available.

#### 1.2 Experimental animals

The animals used in the study were male Dunkin Hartley guinea pigs (300∼500 g) obtained from Orient Bio Inc. (Seongnam, Korea). After obtaining the guinea pigs, the homogeneous inbred animals were given a week to acclimate to their environment. They were housed in temperature- (23±3°C) and light/dark cycle (12/12 h)-controlled rooms with standard rodent food and water available *ad libitum*. All of the animal experiments were approved by the Institutional Animal Care and Use Committee of the Catholic University of Daegu (Daegu, Korea) (Approval Number: IACUC-2009-002).

#### 1.3 Tube insertion and electrode connection

Guinea pigs were anaesthetised by intraperitoneal injection of pentobarbital at a dose of 30 mg/kg, and then were affixed to test beds. Polyethylene tubes (PE-10 tube) were inserted into the jugular vein and the carotid artery. The electrodes for ECG measurement were fixed near both armpits, and earth terminals were attached to the abdomen.

#### 1.4 Administration of sparfloxacin

Through an infusion pump connected to the jugular vein, sparfloxacin was injected into each animal at the rate of 2 ml/h for 1 h. Sparfloxacin was administered at doses of 33.3, 100, and 300 mg/kg.

#### 1.5 Collection of blood samples

Plasma samples for measuring endogenous metabolites were prepared by collecting 1 ml blood from the jugular vein 1 h before and after the administration of sparfloxacin. Each sample was placed into a heparin-treated tube and centrifuged (13,200 rpm; 10 min, 4°C) to separate plasma. The plasma samples were stored in a deep freezer (−70°C) immediately after centrifugation.

#### 1.6 ECG analysis

**Figure 2:**
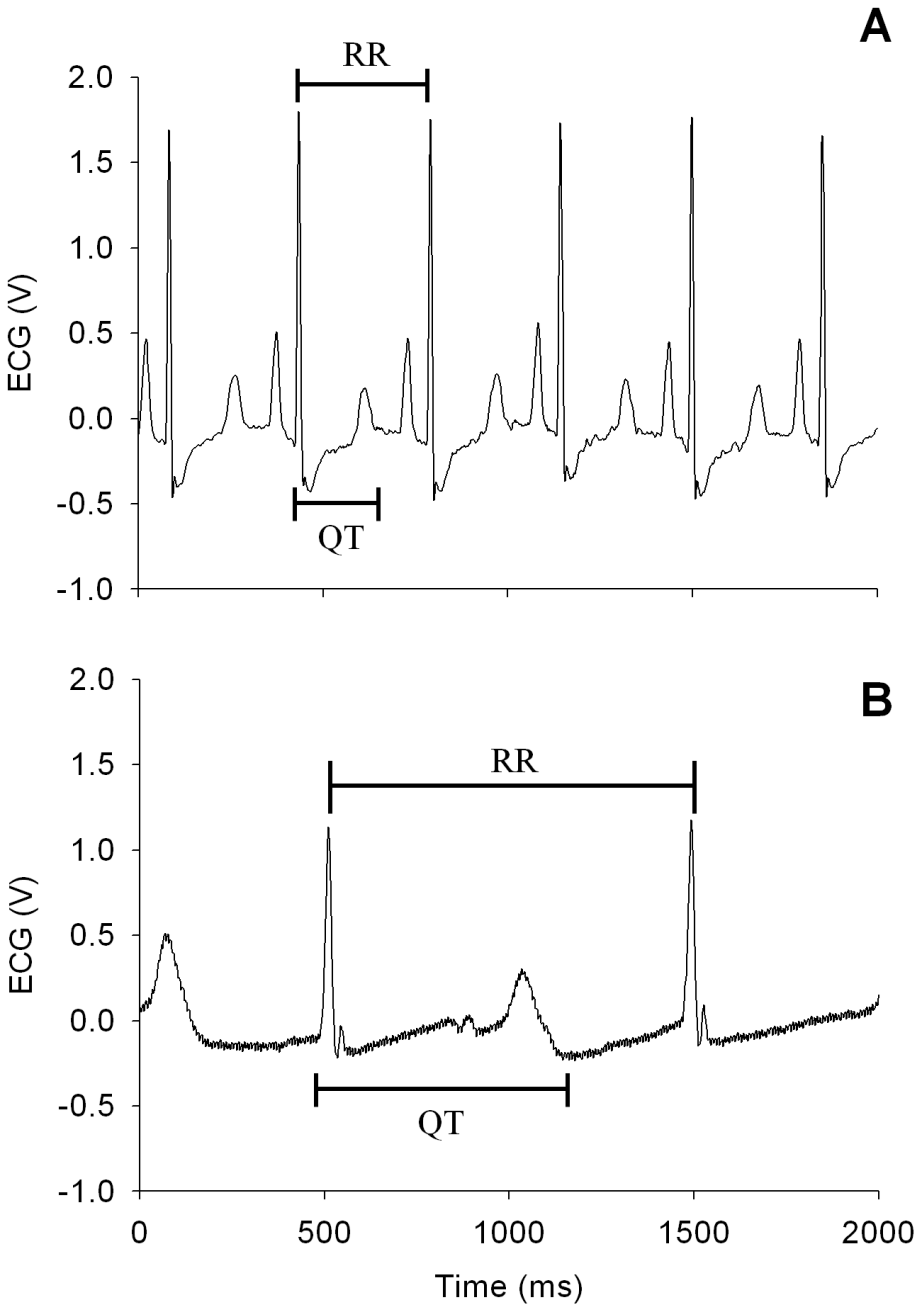
Guinea pig electrocardiograms before (A) and after (B) the administration of sparfloxacin.

Actual measurement of the QT and RR intervals was conducted using ECG measurement equipment (Bio-Amp®, PhysioLab, Korea). The QT interval that takes heart rate into consideration (QTc) was calculated using Bazett's formula [48], [49], [50], as follows: QTc = QT/(RR')^1/2^ (Figure 2). ECG measurements were continued until the final blood sample was collected, while sampling of the ECG data was performed in 10-min intervals from the time the drug was administered until the experiment was completed (for 3 h). At each time point, 20 ECG waves were selected to calculate the mean QTc value, which was represented as the percentage of the interval measured before administration of sparfloxacin.

#### 1.7 Analysis of sparfloxacin concentrations

Plasma concentrations of sparfloxacin were quantified using an HPLC system (Applied Biosystems, Foster City, CA, USA). The compounds were separated on a reversed-phase column (Zorbax 300 SB-C18; 4.6×250 mm inner diameter; 5 µm particle size; Agilent Technologies, Santa Clara, CA, USA) with a Security Guard cartridge (4×2.0 mm inner diameter; Phenomenex, Torrance, CA, USA). The mobile phase was a mixture (55∶45, v/v) of methanol and 0.1% triethylamine solution adjusted to pH 3.0 with perchloric acid and was prepared daily. The column temperature was 40°C, and the flow rate was 1.0 ml/min. Aliquots of thawed plasma (30 µl) were vortex-mixed with methanol and 150 µl 0.1% triethylamine solution adjusted to pH 3.0 with 3 µl perchloric acid as in internal standard for 30 s. After centrifugation at 13,200 rpm for 10 min, the supernatant was transferred to an injection vial. The calibration curve (5–400 µg/ml) and quality-control samples were prepared in drug-free plasma and analysed in the same manner daily before analysis. Samples with concentrations above the calibration curve range were subsequently diluted with drug-free blank plasma and re-analysed to confirm concentration accuracy. The inter-day (n = 5) and intra-day (n = 3) coefficients of variation for this assay were <10% and the correlation coefficient (r) for the calibration curve was >0.999.

### 2 LC–MS-based global metabolomic profiling

#### 2.1 Sample preparation

Plasma samples of 100 µl were diluted in 300 µl methanol for protein precipitation. Then they were vortexed at 2,000 rpm for 5 min and centrifuged (13,200 rpm, 5 min, 4°C). The supernatant (150 µL) was transferred to a vial and 5 µL samples of this solution were injected into the LC–MS system for analysis.

#### 2.2 LC–MS analysis for metabolic profiling

The LC–MS-based global or non-targeted metabolic profiling methodology performed is described elsewhere [29], [38], [51]. Each of the test samples was analysed by LC–MS in the positive ionisation mode to obtain metabolite profiles. The analysis involved the following steps: plasma sample preparation, full-scan (50–900 *m/z*) LC–MS analysis, data preprocessing, peak detection and alignment (using XCMS software) [52], [53], peak intensity normalization (using the quantile normalization algorithm of the preprocessCore package [54] with R language, version 2.11.1) [55], and the creation of an export annotated peak data table along with unique peak identifiers and normalised peak intensities for further multivariate statistical analysis.

To avoid systematic instrument-caused variation in analysis, all samples were randomized prior to LC–MS analyses, and pooled quality-control samples (prepared by mixing equal volumes of all samples) were incorporated. Specific quality-control samples were used to monitor the performance of the method. Peaks with unacceptable variation in quality-control samples (CV>20%) and peaks corresponding to the *m*/*z* of the drugs were excluded from the exported peak data table (obtained using the XCMS software) before further normalization and statistical analysis. Blank chromatograms were acquired by injecting solvent mixture at frequent intervals during analysis to check background noise and sample carryover effects, and they were used to subtract background noise from sample chromatograms. These peaks present in blank samples and identified as noise signals were also excluded before normalization and statistical analysis.

### 3 PCA and PLS analysis

#### 3.1 Software

The SIMCA P+ (version 12; Umetrics, Umeå, Sweden) software package was used for all computations related to PCA and PLS multivariate analyses.

#### 3.2 Validation of the PCA model

PCA is an unsupervised pattern-recognition method. PCA was performed to reveal the general clustering, grouping, and trends among the subjects. The first principal component (PC) (t[1]) represents the most variance in the data. The second PC (t[2]) is orthogonal to t[1]. PCA produces a simpler representation of data and reduces the number of variables that need to be considered. The loadings for each PC describe its multivariate make-up as a vector in the multivariate space. Thus, these loadings identify the underlying variables that are important to each PC.

#### 3.3 Validation of PLS model

Validation of the PLS model was performed using two methods: cross-validation using the leave-out approach (exclusion of 1/7^th^ of the dataset each time), and internal validation using 20 permutation tests and 100 permutation tests, followed by a comparison of the resulting goodness of fit (R^2^) and predictive ability Q^2^ values. Internal validation of the PLS model was performed by randomly changing the order of Y data 20 times in relation to X data to generate 20 separate models that were fit to all permuted Y values with two latent variables. 100 permutation tests were performed for a stricter validation criterion. For this model to be valid, all permuted R^2^ and Q^2^ values should be smaller than the values of the PLS model, and the regression line of the Q^2^-points should intersect the Y-axis at or below zero.

### 4 Metabolite identification

Metabolite identification was performed for the most significant (variable importance to the projection [VIP]>1.5) metabolite ions from the PLS model. By analysing pooled plasma samples, LC–MS/MS scans of selected metabolite ions were acquired, with consideration of their retention times. Next, the obtained data were searched for potential metabolites using relevant literature and online databases such as the Human Metabolome Database (HMDB, http://www.hmdb.ca), MassBank (http://www.massbank.jp/), Metlin (http://metlin.scripps.edu/), and Lipid Maps (http://www.lipidmaps.org/). Similarly, MS/MS and retention time data from commercial standards of those potential metabolites were acquired and compared to the selected metabolite ions to confirm the identification. Metabolites with no easily available commercial standards were identified putatively by interpreting the fragmentation patterns of metabolite ions and comparing our data to the available metabolite databases (mentioned above) and the literature. Due to inherent limitations of the low-resolution LC–MS technique and limited available metabolomic resources, we were unable to identify all of the selected metabolites.

### 5 Metabolic network analysis

A hypothetical metabolic network was organized using Cytoscape (version 2.6.2) and was constructed by extracting data from the Kyoto Encyclopedia of Genes and Genomes (KEGG) [56], HMDB, Lipid Maps [57], and MetaCyc [58] pathway databases, as well as from the literature [59]. The node size indicates the identified metabolites (large) and their neighbours (small). The nodes are colour-coded according to their respective modules as follows: steroid-related metabolism (pink); pyrimidine metabolism (sky blue); glycerophospholipid metabolism (yellow); alanine, aspartate/glutamate metabolism (orange); fatty acid metabolism (green); and pentose-related metabolism (apricot). The edges represent metabolic reactions (arrows); indirect or possible reactions involving several intermediates are located between the connected nodes. All of the abbreviations used for enzymes, genes, and reactions were from KEGG identifiers (http://www.genome.jp/kegg/kegg3.html).

### 6 Comparison of the significant differences in the intensity of key metabolites between the groups

A normality test was necessary because the number of identified metabolites did not exceed 30. ANOVA, a parametric method, was used if the p-value was larger than the significance level of 0.05 by the Kolmogorov-Smirnov test, because normality was satisfied. The Kruskal-Wallis test, a non-parametric method, was used if the p-value was smaller than the significance level of 0.05 by the Kolmogorov-Smirnov test, because normality was not satisfied. Statistical analyses were performed using the SPSS software (version 12.0 for Windows; SPSS, College Station, TX, USA). Differences were considered statistically significant at p<0.05.

## Results

### 1 Metabolic profiling of plasma samples

Global metabolomic profiling, which involves the collective analysis of metabolites, is an essential technique for discovering metabolic phenotypes that can predict individual QT prolongation. According to previous studies [60], [61], LC–MS analysis can provide large amounts of information about metabolites from small samples, with high sensitivity to molecules of a broad range of molecular weights. LC–MS analysis was performed on the plasma samples collected to generate global metabolic profiles from the 15 guinea pigs after administration of sparfloxacin at doses of 33.3, 100, and 300-mg/kg (Figure S1). The peaks of the endogenous metabolite except not only sparfloxacin but also the drug metabolites were processed (Figure 1). Then the XCMS software was used to detect 1,178 common metabolic features(i.e., peaks) from the 15 datasets. The major peaks detected corresponded to steroids, lipids and pyrimidines, as well as a broad range of molecules including other metabolites.

Finally, the intensities of the 1,178 peaks were normalised using a quantile normalization algorithm [55] to remove systematic errors from sample preparations and LC–MS analyses. The peak intensities represent a metabolic phenotype for each sample, which varied among samples and thus represented the individual variation in the plasma metabolome.

### 2 ECG analysis

**Figure 3:**
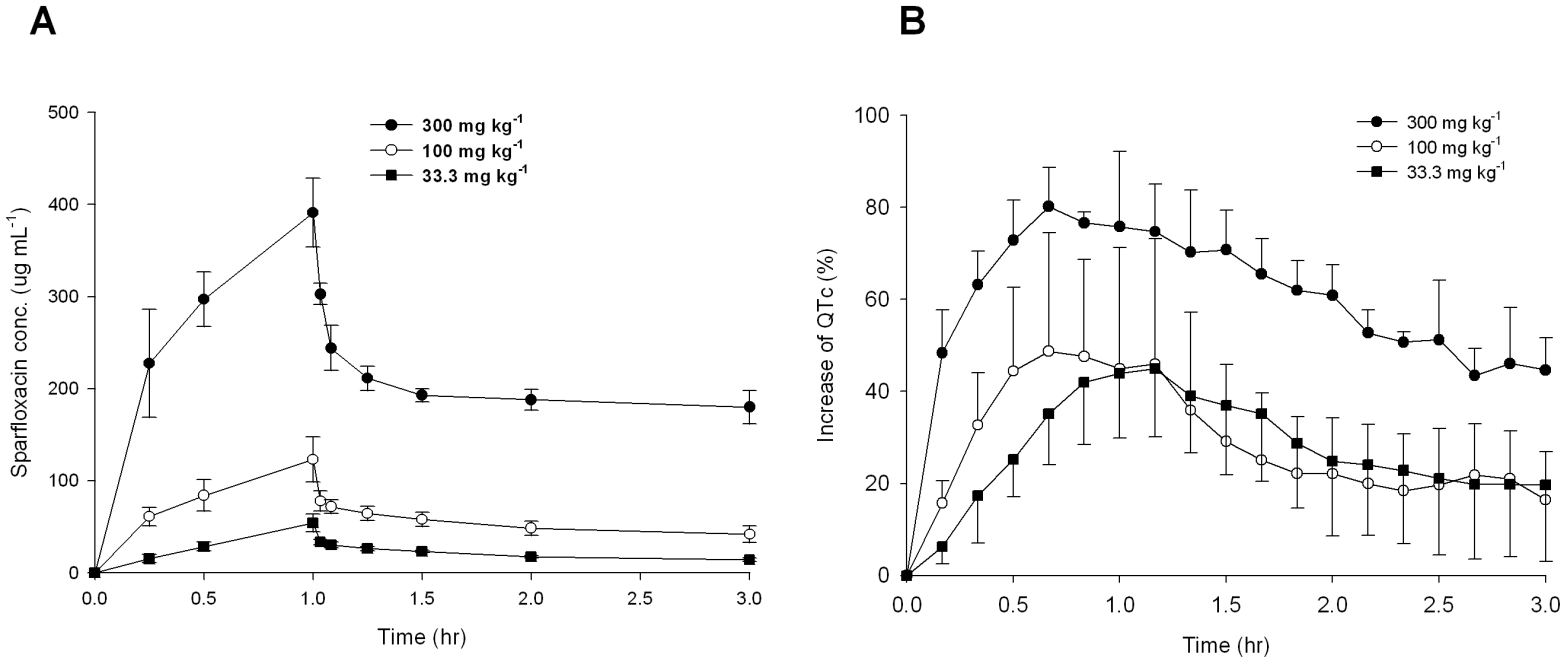
Mean plasma concentration and mean increase in QTc (%) over time according to sparfloxacin doses. (A) Mean plasma concentration of sparfloxacin following a single 1-h intravenous dose of 33.3 mg kg^−1^, 100 mg kg^−1^, or 300 mg kg^−1^. (B) Mean increase in QTc (%) following a single intravenous dose of 33.3 mg kg^−1^, 100 mg kg^−1^, or 300 mg kg^−1^. The percentage QT increase was less in the group dosed with 100 mg kg^−1^ than that with 33.3 mg kg^−1^ after 1 h. Bars indicate standard deviations.

To evaluate QT prolongation from the investigated guinea pigs, the mean QTc value was represented as the percentage, using the interval measured before administration of sparfloxacin (control) as the baseline. As shown in Figure 3, an increase in drug dose resulted in an increase in both plasma sparfloxacin concentration and the percent change in QT interval. This made it possible to detect drug toxicity based on QT prolongation, and also to determine the metabolite that exerts the strongest effect on QTc values. Thus, the percent change in QT interval was chosen as a response variable for the PLS analysis.

### 3 Prediction of individualised QT prolongation

**Figure 4:**
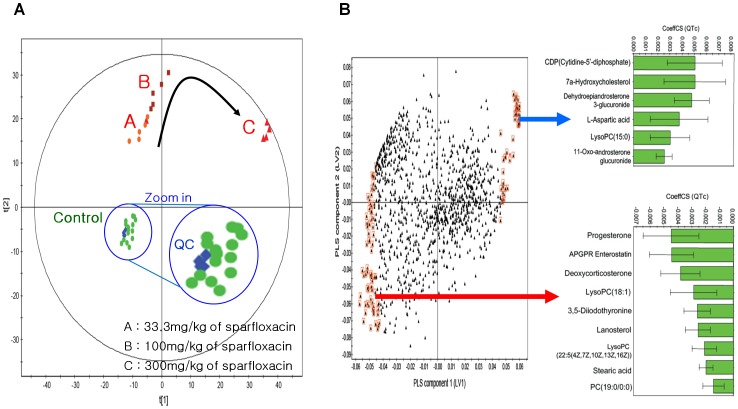
PCA and PLS modelling of plasma LC–MS metabolic data for predicting the drug-induced QT prolongation of sparfloxacin. (A) PCA score plot (t[1] vs. t[2]) obtained from guinea pig plasma samples. Obviously separated clustering of dose groups and the control group was shown by PCA; in addition, dose-dependent metabolomic modification was detected. (B) Loading plot for the above PLS model in which each point represents a metabolic feature detected from plasma LC–MS data and is plotted as its respective coefficient from PLS component 1 vs. its coefficient from PLS component 2. The arrow indicates a positive relationship with the QTc. Metabolite variables with larger coefficient values (positive or negative) have a stronger correlation with the QTc (marked by red boxes; VIP>1.5) and were used to build the PLS model for predicting cardiovascular toxicity. The inset green bar plot shows the correlation coefficients for the key identified metabolites.

To build a statistical model that can predict individualised QT prolongation effectively using the measured metabolic data, we employed PCA and PLS analysis. The pool sample quality-control data can be examined visually for gross changes to provide a rapid assessment of how well the run performed. Similarly, a small number of selected components can be rapidly screened for peak shape, intensity, mass accuracy, and retention time against predetermined acceptance criteria. Assuming that these criteria are met, the entire data set can be used for initial multivariate statistical analysis by PCA. As shown in Figure 4A, all of the quality-control data (blue colour) clustered closely together. The PCA score plots for the 1,178 peak intensities (X block) are also shown in Figure 4A. The plots show distinct grouping patterns for control and drug-dose (low, middle, high) groups from the plasma model (3 PCs, R^2^ = 0.687, Q^2^ = 0.536).

PLS is a multivariate statistical analysis that can build an efficient model using X variables (metabolites) to predict Y variables (QTc) via reduction of dimensionality. Such dimension reduction is achieved by generating several PLS components (also called latent variables [LVs], each of which is a linear combination of metabolites in the X block) [62] that maximise the correlation between the LVs and the QTc. Subsequently, the prediction model can be built by relating the LV values to the QTc values (see below). We performed PLS analysis for all 1,178 peak intensities (X block) to the QTc (Y block) to predict QT prolongation. The first two LVs were included in the PLS model based on their eigenvalues and Q^2^ statistics. Based on this model, we selected the metabolites (X variables) that made a large contribution to predicting QT prolongation (Y variable-QTc).

Figure 4B shows the coefficients (PLS loadings) of the 1,178 variables in the linear combinations for the first two LVs. A high coefficient value in the first LV indicates that the corresponding metabolite made a large contribution to the prediction of QT prolongation using QTc values. A positive coefficient means that the corresponding metabolite has a positive relationship with the QTc; negative coefficients indicate an inverse relationship. To focus on metabolites associated with QTc, a set of metabolites was selected that made a large contribution to its prediction using VIP measurements, which represent the collective contribution of the individual metabolites from the first two LVs to QT prolongation prediction. The 106 metabolites selected (VIP>1.5) are enclosed by red boxes in Figure 4B.

**Figure 5:**
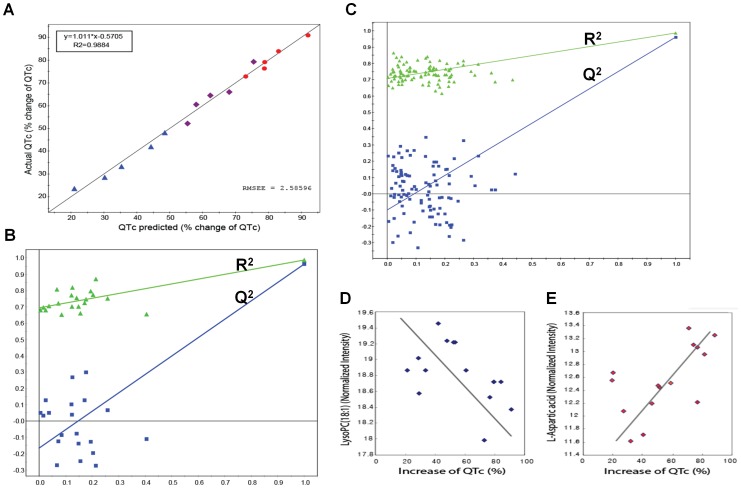
PLS model validity. (A) Plot of predicted QTc vs. actual (measured) QTc from the PLS model using the cross-validation method. Predicted values from the PLS model in which all predicted QTc values show a linear relationship with actual measured QTc values (R^2^ = 0.9884). Colour from blue to red indicates increasing QTc values. RMSEE specifies the root mean square error of the estimation (the fit) for observations in the workset. The values were predicted by exclusion of 1/7^th^ of the data from the model and predicting the excluded data that are not part of model building. (B) Internal validation of the PLS model by 20 permutation tests to confirm predictability and data overfitting shows that all R^2^ (goodness of fit) and Q^2^ (predictability of model) values from the permuted models (left) are smaller than those of the original model (far right), demonstrating the validity of the PLS model. (C) Internal validation of the PLS model with 100 permutation tests to use stricter validation criteria. (D and E) Plots for normalised intensities of LysoPC (18∶1) (D) and L-aspartic acid (E), which exhibit a negative and positive correlation, respectively, with QTc.

This model included two PLS components (LVs) that had high eigenvalues (10.3 and 4.5 for the two LVs, respectively), goodness of fit (R^2^ = 0.994), and high predictability (Q^2^ = 0.924) estimated by leave-one-out cross-validation (LOOCV) experiments [63]. All predicted QTc values from this model showed excellent correlations with actual measured QTc values, as indicated by the regression line of 0.9884 for the predicted versus actual QTc plot (Figure 5A). Internal validation was also performed with this PLS model to check its ability to predict new observations with no risk of overfitting the data and capturing non-systematic correlations between the metabolites and QTc values. The goodness of fit (R^2^) and predictability (Q^2^) measures from 20 and 100 random permutation experiments [64] are shown in Figure 5B and 5C. All R^2^ and Q^2^ values from the permutation experiments (left) were smaller than those of the PLS model (far right). Additionally, the negative intercept (−0.16 in Figure 5B; −0.11 in Figure 5C) of the Q^2^ regression line indicated no data overfitting in the model. Guinea pigs were used in this study to avoid the risk of high-dose administration in humans, while the sample size was increased to the minimal sample size that would allow prediction of QT prolongation in the guinea pig according to dose change (33.3, 100, 300 mg/kg), and the validity of the stricter model was established based on 100 random permutation tests.

Finally, we selected a set of 106 key metabolic features characterising individualised QT prolongation, represented by the QTc as those with VIP>1.5 in the PLS model. Interestingly, some of these selected metabolites displayed positive correlations with the QTc, while others mainly exhibited negative correlations. The correlation coefficients between some of the selected metabolites and the QTc are shown in Figure 4B (green bars). Strong correlations were found between two key metabolites, LysoPC (18∶1) and L-aspartic acid, versus the QTc values in the 15 samples (Figure 5D and E).

### 4 Identification of key metabolites associated with individualised QT prolongation

An LC–MS/MS-based (tandem mass spectroscopy) method was used to recognise the biological importance of 106 selected metabolites in individualised QT prolongation. To obtain information about the 106 MS fragment patterns and retention times using LC–MS/MS, a pooled plasma sample was analysed, prepared by mixing together an equal amount of each plasma sample. Molecular weights of parents were measured, and daughter ions were obtained by the MS/MS technique (Figure S2a). Adducts (e.g., Na, K) of expected candidate metabolites were considered, and candidate metabolites were identified by HMDB [65] using a *m/z* tolerance of 0.5-Da (Figure S2b). Next, we analysed commercial standards for candidates using the same LC–MS/MS method (Figure S2c) and matched their retention times and MS/MS data to those of the selected parent ion. The same method was applied to identify parent ions of the remaining selected key metabolites. When commercial metabolite standards were not easily available, we identified the corresponding metabolites using database searches and other resources. Table 1 summarises the 15 metabolites identified and relevant metabolic pathways. The 10 metabolites marked with asterisks were identified using commercial standards, while the other metabolites were identified based on database searches and literature reports.

**Table 1:** Names and associated metabolic pathways for the identified metabolites in increasing order of their VIP values.

Identification	VIP^a^	*m/z* of parent ion (MS)	HMDB ID^**^	Pathway/Process or Class	Reference
LysoPC(15∶0)*	1.57	482	HMDB10381	Glycerophospholipid metabolism	KEGG Pathway (ko00564)
LysoPC(18∶1)*	1.59	522	HMDB02815	Glycerophospholipid metabolism	KEGG Pathway (ko00564)
LysoPC(22∶5(4Z,7Z,10Z,13Z,16Z))	1.61	570	HMDB10402	Glycerophospholipid metabolism	KEGG Pathway (ko00564)
PC(19∶0/0∶0)	1.51	538	-	Phospholipid metabolism	HMDB class and LMGP01050041
Lanosterol*	1.61	427	HMDB01251	Steroids and Steroid Derivatives	KEGG Pathway (ko00100)
Progesterone*	1.61	315	HMDB01830	Steroids and Steroid Derivatives	KEGG Pathway (ko00140)
Deoxycorticosterone*	1.69	331	HMDB00016	Steroids and Steroid Derivatives	KEGG Pathway (ko00140)
7a-Hydroxycholesterol*	1.96	403	HMDB01496	Steroids and Steroid Derivatives	KEGG Pathway (ko00120)
Stearic acid*	1.53	285	HMDB00827	Fatty acid biosynthesis	KEGG Pathway (ko00061)
3,5-Diiodothyronine*	1.62	526	HMDB00582	Amino acids	HMDB class and ref.
CDP(Cytidine-5′-diphosphate)*	1.97	404	HMDB01546	Pyrimidine metabolism	KEGG Pathway (ko00240)
APGPR Enterostatin	1.62	497	HMDB06117	Polypeptides	HMDB class and ref.
L-Aspartic acid*	1.65	134	HMDB00191	Alanine, aspartate, and glutamate metabolism	KEGG Pathway (ko00250)
11-Oxo-androsterone glucuronide	1.57	481	HMDB10338	Glucuronides	KEGG Pathway (ko00040)
Dehydroepiandrosterone 3-glucuronide	1.59	465	HMDB10348	Glucuronides	KEGG Pathway (ko00040)

* Metabolites were identified by interpreting their fragmentation patterns (MS/MS spectra) and conducting a database search. LysoPC, lysophosphatidylcholine; PC, phosphatidylcholine; CDP, cytidine-5′-diphosphate; APGPR, Ala-Pro-Gly-Pro-Arg. ^**^Human Metabolome Database. ^a^Variable importance in the projection. All abbreviations used for pathways and reactions are from KEGG identifiers (http://www.genome.jp/kegg/kegg3.html).

### 5 Prediction of the potential functional association of key metabolites with individualised QT prolongation

**Figure 6:**
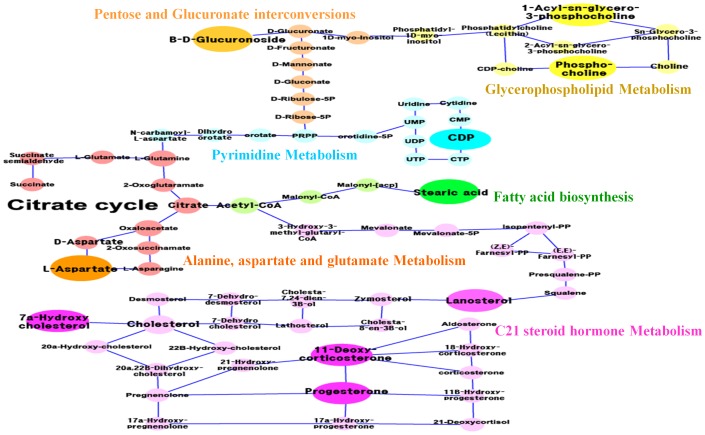
Metabolic network for 15 identified metabolites. The large nodes in the network represent the key identified metabolites, while the small nodes represent their neighbours in the respective metabolic pathways (see Table 1). Metabolic reactions (arrow) and indirect or possible reactions involving several intermediates between the connected nodes are indicated. This metabolic network revealed six major modules, shown in different colours. All abbreviations used for enzymes or genes and reactions are from KEGG identifiers (http://www.genome.jp/kegg/kegg3.html).

To investigate the potential functional roles of the metabolites identified in individualised QT prolongation, a hypothetical metabolic network (Figure 6) was reorganised using the 15 identified metabolites (Table 1) and their first neighbours from the metabolic interactome obtained from the KEGG databases [56], Lipid Maps [57], MetaCyc [58], and HMDB, as well as the literature reports [59]. The node size indicates the identified metabolites (large) and their neighbours (small). The edges represent metabolic reactions (arrows); indirect or possible reactions involving several intermediates are located between the connected nodes. The six major network modules (different node colors) are presented in Figure 6; steroid, pyrimidine, glycerophospholipid, alanine, fatty acid, and pentose-related metabolism (Table 1).

The reactions associated with the selected metabolites in the major modules may have important effects on the cardiac toxicity of sparfloxacin, thus acting as determinants of the individualised QT prolongation of sparfloxacin. Interestingly, lipids (e.g., lysophospholipid, phosphatidylcholine [lysoPC]) have been reported to cause endothelial vasomotor dysfunction in isolated blood vessels [66]. Diabetes is positively associated with the proportion of stearic acid in plasma phospholipids [67]. In addition, altered levels of fatty acids such as stearic acid suggest a disrupted pathway of choline metabolism [68]. In the present study, a decrease in lysoPC(18∶1) tended to result in a decrease in stearic acid. According to another report, intravenous (i.v.) administration of cytidine-5′-diphosphate choline (CDP-choline) (100, 250, and 500 mg kg^−1^) increases blood pressure [69]. Figure 4B shows that there was a strong correlation between CDPs with larger coefficient values and the QTc value. DOCA/salt treatment modifies cardiac electrical activity (i.e., increased arrhythmogenic activity and prolongation of PR and QTc intervals) in one- and two-renin gene mice [70], and progesterone levels in women are inversely correlated with ibutilide-induced QT interval prolongation [71]. Figure 4B shows that, in the present study, progesterone had larger negative coefficient values, confirming that progesterone is a marker that affects QT interval prolongation. In addition, the selected metabolites with negative relationships with the QTc (Figure 4B) mostly belonged to the same steroid module in the network, indicating the validity of the selected metabolites regarding their functional association with QT prolongation. Nonetheless, most of the other selected metabolites have never been reported to be associated with the cardiac toxicity of sparfloxacin, suggesting their potential as novel determinants of the individualised QT prolongation of sparfloxacin.

### 6 Comparison of the significant differences in the intensity of key metabolites between the groups

**Figure 7:**
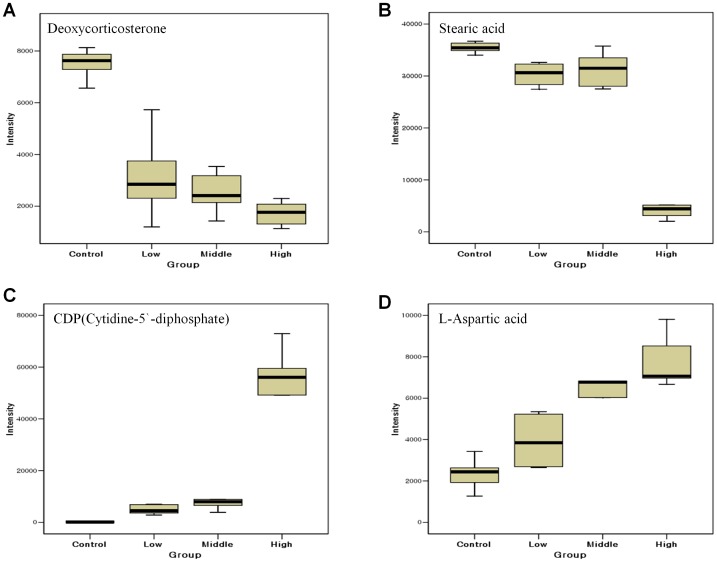
Comparison of the distribution of metabolite intensity levels for control and drug-dosed (low, middle, high) groups. Box plots indicate the distribution of magnitudes of peak intensity levels of key metabolic phenotype in each group. The box is drawn from the 25^th^ to 75^th^ percentiles in the distribution of intensities. The median, or 50^th^ percentile, is drawn as a black horizontal line inside the box. The whiskers (lines extending from the box) describe the spread of the data within the 10^th^ and 90^th^ percentiles.

Deoxycorticosterone, lanosterol, 3,5-diiodothyronine, L-aspartic acid, lysoPC(15∶0) and lysoPC(18∶1) satisfied normality, because their p-values were larger than the significance level of 0.05 by the Kolmogorov-Smirnov test. ANOVA was carried out with the metabolites identified that satisfied the normality test. Significant differences in peak intensity levels were seen between the control, low dose, middle dose, and high dose groups. In the cases of deoxycorticosterone, lanosterol, 3,5-diiodothyronine, L-aspartic acid and lysoPC(15∶0), the p-value of the peak intensity between the dosed and control groups was <0.001, whereas that of lysoPC(18∶1) was 0.016. Progesterone, 7a-hydroxycholesterol, stearic acid and cytidine-5′-diphosphate (CDP) did not satisfy normality, because their p-values were smaller than the significance level of 0.05 by the Kolmogorov-Smirnov test. The Kruskal-Wallis test was used with the metabolites that failed to satisfy the normality test. As in the cases of the metabolites that satisfied the normality test, there were significant differences in peak intensity between the control, low dose, middle dose, and high dose groups. In the cases of progesterone, 7a-hydroxycholesterol, stearic acid and CDP, the p-values of the peak intensity between the dosed and control groups were <0.001. CDP, deoxycorticosterone, stearic acid and L-aspartic acid were selected as key metabolic phenotypes that would be able to reflect QT prolongation well in clinical settings, and their peak intensities are presented in a box plot by group (Figure 7). In the case of deoxycorticosterone, the p-values of the peak intensity between the low dose and control groups, between the middle dose and control groups and between the high dose and control groups were all <0.001 (Figure 7A). In the case of stearic acid, the p-values of the peak intensity between the low dose and control groups, between the middle dose and control groups and between the high dose and control groups were all <0.001, while that between the middle dose and high dose groups was 0.004 (Figure 7B). In the case of CDP, the p-values of the peak intensity between the low dose and control groups, between the middle dose and control groups and between the high dose and control groups were all <0.001, while that between the low dose and high dose groups was 0.001,and that between the middle dose and high dose groups was 0.031 (Figure 7C). In the case of L-aspartic acid, the p-values of the peak intensity between the middle dose and control groups and between the high dose and control groups were <0.001, while that between the low dose and middle dose groups was 0.008, and that between the low dose and high dose groups was <0.001 (Figure 7D).

### 7 Selection of a clinically applicable metabolic phenotype predictive of individualised QT prolongation

A metabolic phenotype associated with 15 metabolites would still be complicated to use for predicting the QT prolongation of sparfloxacin in clinical practice. Thus, we reduced the profile further by integrating all the findings, including the key metabolites selected from the PLS model, the six major modules in the network, and the contribution of each metabolite to the prediction of the QTc value (i.e., VIPs), and also considering the convenience of their use in a clinical setting. This integrative approach allowed us to select the following four metabolites, readily applicable in the clinical setting, from the six network modules: CDP (CDP; VIP = 1.97), representing pyrimidine metabolism; deoxycorticosterone (DC; VIP = 1.69), representing steroid-related metabolism; stearic acid (SA; VIP = 1.53), representing fatty acid biosynthesis metabolism; and L-aspartic acid (LA; VIP = 1.65), representing alanine, aspartate, and glutamate metabolism.

**Figure 8:**
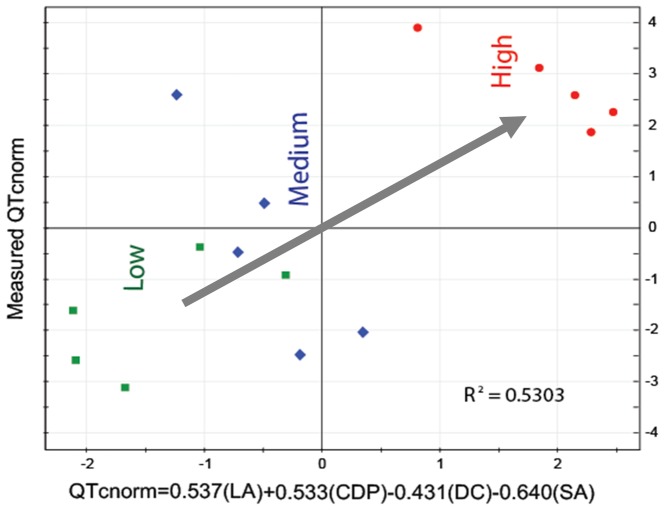
Scatterplot of the predicted normalised QTc values (QTc_norm_) from the equation QTc_norm_ = 0.537(LA)+0.533(CDP) – 0.431(DC) – 0.640(SA), versus the measured and normalised (QTc_norm_) values for the 15 samples. Using this prediction index with only four metabolite abundances, subjects can be categorised into low, medium, and high QTc groups.

Recently, the use of pathways has been demonstrated to outperform individual molecules for predicting disease subtypes [72]. Hence, we hypothesised that the four metabolites representing the major network modules and their associated pathways could be used collectively to improve the prediction of individualised QT prolongation. We applied PLS analysis to the four metabolites and developed a measure that can be used to predict the cardiac toxicity of sparfloxacin from the resultant PLS model (considering the coefficients from the correlation structure of these four metabolites with the QTc and their normalised intensities in test samples) as follows: QTc_norm_ = 0.537LA+0.533CDP−0.431DC−0.640SA. A scatterplot of the predicted versus measured QTc values for the 15 samples using this equation is presented in Figure 8. The prediction power (R^2^ = 0.5303) suggests that the prediction capability is reasonably well. As shown in Figure 8, the prediction equation can also be used to categorise the subjects into three cardiac toxicity groups (high, medium, and low QTc groups) based on their plasma levels of these four metabolites. To prove the validity of prediction equation, we re-established a predictive model and a predictive equation using 12 guinea pigs by the same approach, and then predictive accuracy was confirmed by applying the predictive equation to the other 3 guinea pigs (Figure S3, S4 and Table S1, S2). Thus, the use of the four metabolites allows us to predict the individualised QT prolongation of three different doses of sparfloxacin, and monitor the activities of the key metabolic pathways affecting the pharmacodynamics of sparfloxacin in clinical applications.

## Discussion and Conclusions

Endogenous metabolites in the human body may change due to many factors, such as dietary habits, the environment, heredity, disease and medicines. We developed a pharmacometabolomic approach to discover metabolic phenotypes that could predict changes in biochemical metabolites directly related to physiological or pathological functions and that can also predict drug toxicity in the guinea pig. Metabolic phenotypes can be applied to understand pharmacological roles related to pharmacodynamics, as well as to pre-clinical or clinical settings. Cardiovascular risk factors are strongly related to abdominal obesity, increased blood pressure, impaired fasting glucose and dyslipidemia, and also increased incidence and lethality of ischemic heart disease and stroke [73], [74], [75]. Although it is known that such factors increase cardiovascular risk [76], [77], [78], [79], little is known about the relationship with asymptomatic risk factors, such as long QT syndrome on an electrocardiogram. QTc interval prolongation is considered a prognostic factor for arrhythmia although its mechanism has not been completely known, since it is a phenomenon accompanying delayed ventricular repolrarisation [80], [81], [82], [83]. QT interval prolongation is a side effect of sparfloxacin, and has been reported to have I_Kr_-blocking ability [22], [23], [24] and to be related to TdP [14]. *hERG*, one of the genes [84] that can cause long QT syndrome, controls an important repolarising current called I_Kr_. Guinea pigs have hERG channels and thus are suitable for measuring the proarrhythmic effect [85], [86], [87]. This integrative approach was applied to predict drug-induced QT prolongation of sparfloxacin. Significant prolongation of the QT interval was observed when sparfloxacin was administered at doses of 33.3, 100, and 300 mg/kg [44], and these doses were used in our experiments. We analyzed plasma samples using LC-MS, and detected 1,178 metabolic features. These plasma metabolic signatures were used to establish PCA and PLS models that could predict the QT prolongation caused by sparfloxacin. Then, based on the PLS model, 106 key metabolic features were selected that were most related to QTc values. LC-MS/MS analysis and database searches were conducted to identify 15 metabolites. The 15 metabolites identified were reorganised into a hypothetical network depicting metabolic pathways. The network revealed six major modules; metabolism related to steroids, pyrimidines, glycerophospholipids, alanine, fatty acids and pentoses. Finally, the results of metabolic profiling, multivariate analyses and the network were used to select four metabolites that could best predict QT prolongation with clinical applicability, and these four metabolites were used to establish a prediction equation for QT prolongation. This aspect ofmetabolic phenotype can be applied in the early stages of drug development to save time by decreasing the possibility of experimental failure caused by toxicity during the course of later research. The prediction equation may be sparfloxacin-specific, in the form of a weighted linear combination of the four selected metabolites. The potential effects of metabolites on the major network modules of Pharmacodynamics may be due to direct biological effects resulting from an interaction between the drug and biological system. In addition to the lipids mentioned, lysoPC [66], DOCA/salt [70], and progesterone [71] are also known to be involved in QT prolongation and can influence the response to sparfloxacin. Sparfloxacin interacts with several steroids, stearic acid, and CDP, supporting the possible functional role of our metabolic network module in predicting sparfloxacin QT prolongation.

Hypertriglyceridemia, hyperglycemia and obesity may affect QTc intervals [88], [89], may be related to changes in the autonomic nervous system of patients with obesity [90], and may affect the QTc interval by increasing oxygen consumption in the body and thus increasing the heart rate [91]. It may also be that in patients with abdominal obesity, insulin resistance and hyperinsulinemia may cause cardiovascular disease, resulting in QTc interval prolongation [92], [93], [94]. In addition, abnormal pathological changes caused by complex diseases or medication could damage the blood stream into cardiac muscle, leading to asymptomatic- or clinical- heart disease, and this in turn could cause QTc prolongation. The key metabolic phenotypes proposed in this study are also related to various complex diseases. Thus, QT prolongation, an adverse effect of a drug, could not be detected in the early stages, and the merit of this study is that the prediction equation for QT prolongation can be set up for use in the clinical setting. We propose that QT prolongation can be detected early using the prediction equation obtained by a pharmacometabolomic approach that comprehensively involves typical clinical assays (e.g., ECG measurement; [49], [50]), simple chromatography technology [95], normalization, multivariate analyses and network technology.

In conclusion, it was confirmed that QT prolongation increased according to increased dosage of the drug. It is difficult, however, to obtain early information about major determining factors of relevant reactions, such as drug dose, and concentration in plasma and at the action site. Accordingly, we proposed a pharmacometabolomic approach to predict drug-induced QT prolongation. By applying this approach, a model was established for predicting adverse cardiovascular effects of sparfloxacin, and key metabolic phenotypes were identified. Application of such an approach could be extended to research on other fluoroquinolone antibiotics and drug toxicity that involve complex reactions in the pre-clinical or clinical setting, and can provide a foundation for understanding the mechanism(s) of their effect(s) in pharmacological efficacy. Nonetheless, the validity of these findings and hypotheses should be tested on a larger, independent group of samples, and our metabolic phenotype should be integrated into subsequent investigations on human subjects.

## Supporting Information

Figure S1 **Liquid chromatography–mass spectroscopy-based metabolic profiling of plasma samples obtained from 15 guinea pigs after administration of sparfloxacin at doses of 33.3, 100, and 300 mg/kg.**
(TIF)

Figure S2 **Metabolite identification of lysoPC(18∶1).** (A) Plasma MS/MS spectra of the *m/z* 522.3 peak at a retention time (RT) of 17.2 min. (B) MS search for *m/z* 522±0.5 Da from the HMDB database resulted in three possible metabolites that were compared for their MS/MS spectra and RT from their standards in Figure S2a. (C) Only lysoPC(18∶1) matched the MS/MS spectra and RT of the plasma spectra (Figure S2a), making it possible to identify the peak at RT 17.2 min and *m/z* 522.2 as lysoPC(18∶1).
(TIF)

Figure S3 **PCA and PLS model validity for predicting drug-induced QT prolongation of sparfloxacin using 12 guinea pig plasma samples (four guinea pigs in each of the low, medium, and high QTc groups).** (A) PCA score plot (t[1] *vs*. t[2]) obtained using the 12 guinea pig samples. We confirmed that the three groups were separated from each other, in the same pattern as in Figure 3A. Eigenvalues were 3.69 and 9.59. Each eigenvalue reflects the dispersion of the corresponding major component. The explanatory powers of t[1] and t[2] were 27.79% and 72.21%, respectively. (B) Plot of predicted QTc *versus* actual (measured) QTc from the PLS model using a cross-validation method. Predicted values from the PLS model in which all predictions of QTc values showed a linear relationship with actual (measured) QTc values (R^2^ = 0.9953). (C) Internal validation of the PLS model by 20 permutation tests to confirm predictability and data overfitting showed that all R^2^ (goodness of fit) and Q^2^ (predictability of model) values from the permuted models (left) were smaller than those of the original model (far right), demonstrating the validity of the PLS model. (D) Internal validation of the PLS model with 100 permutation tests using stricter validation criteria.
(TIF)

Figure S4 **Scatterplot of the predicted normalized QTc values (QTc_norm_) from the equation QTc_norm_ = 0.402(LA)+0.556(CDP)−0.409(DC)−0.601(SA),** ***versus*** **the measured (QTc_norm_) values for the 12 samples (four guinea pigs in each of the low, medium, and high groups).** Using this prediction index with only four metabolite abundance values, subjects can be categorized into low, medium, and high QTc groups.
(TIF)

Table S1 Measured QTc_norm_^a^ values (95% CI), and predicted normalized QTc values (95% CI) from the QTc_norm_^b^ and QTc_norm_^c^ equations for the three guinea pigs not included in the modeling.
(DOCX)

Table S2 Values of the measured QTc (%) calculated by converting the predicted normalized QTc values from the QTc_norm_^b^ and QTc_norm_^c^ equations for the three guinea pigs not included in the modeling.
(DOCX)
