# Supplementary figures and images for: Pharmacometabolomic Approach to Predict QT Prolongation in Guinea Pigs
Source: PLoS One. 2013 Apr 4;8(4):e60556. doi: 10.1371/journal.pone.0060556 (PMC3617128; doi:10.1371/journal.pone.0060556)

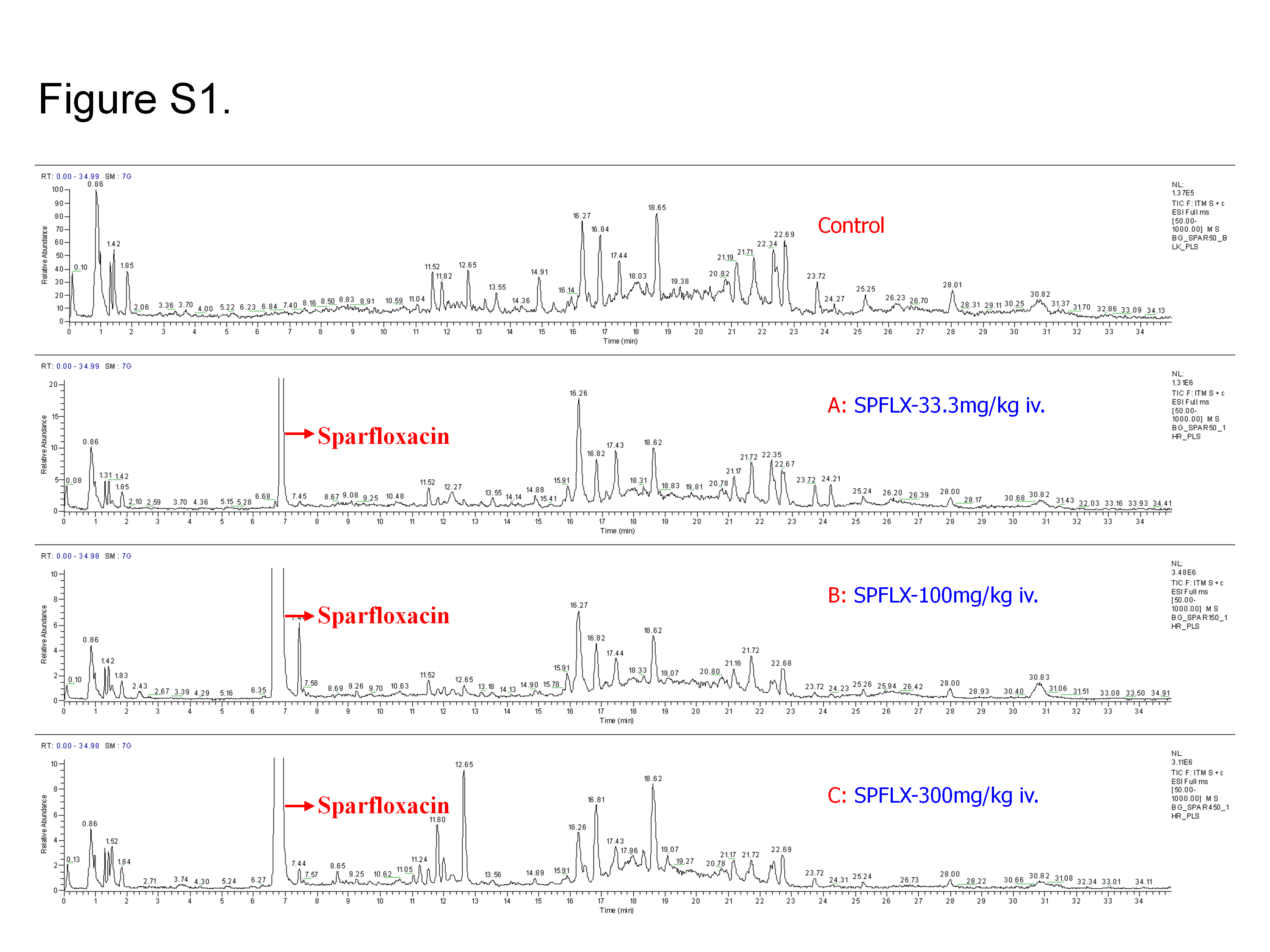

Supplement: Figure S1 — Liquid chromatography–mass spectroscopy-based metabolic profiling of plasma samples obtained from 15 guinea pigs after administration of sparfloxacin at doses of 33.3, 100, and 300 mg/kg. (TIF) [file pone.0060556.s001.tif]

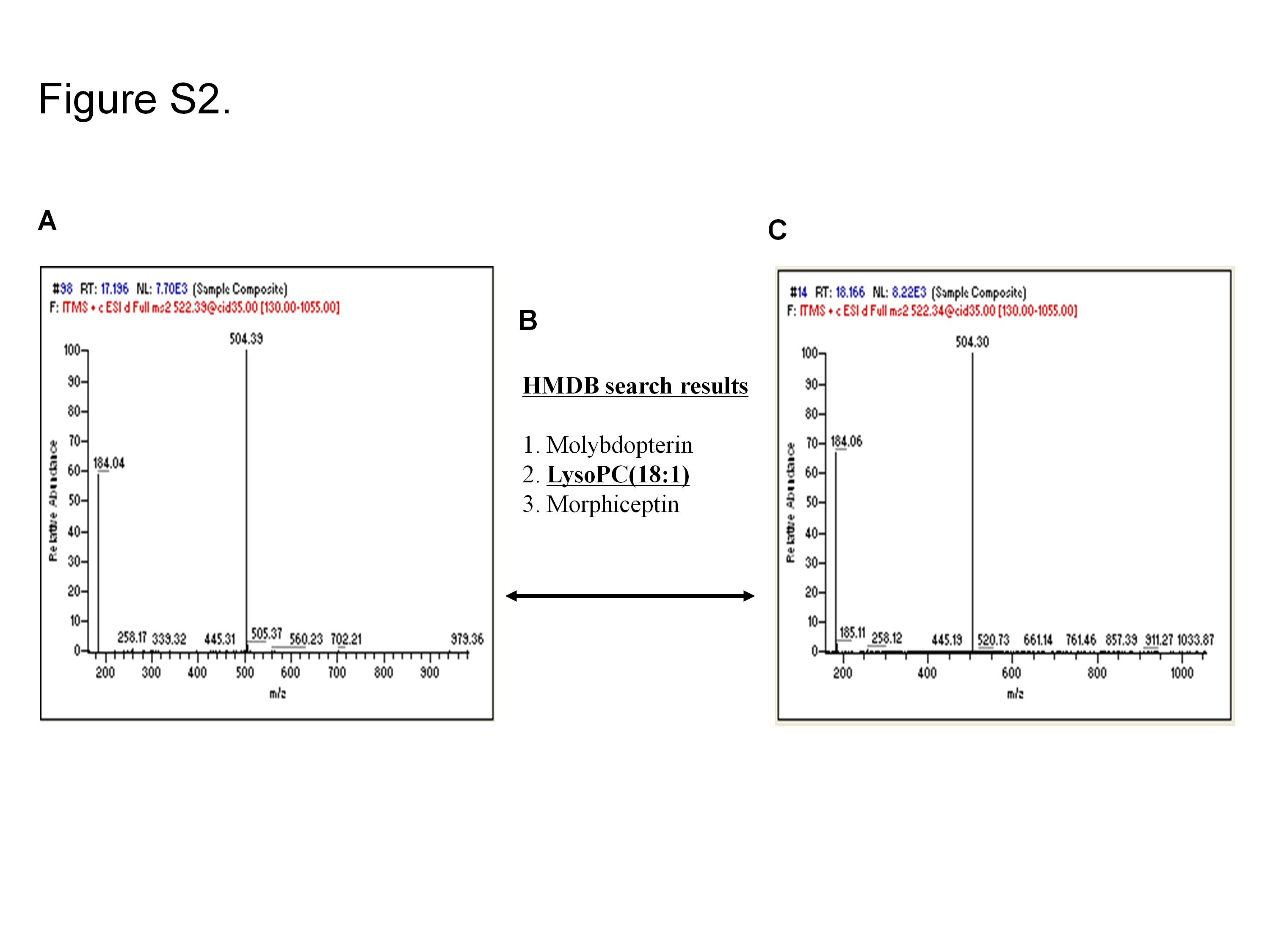

Supplement: Figure S2 — Metabolite identification of lysoPC(18∶1). (A) Plasma MS/MS spectra of the m/z 522.3 peak at a retention time (RT) of 17.2 min. (B) MS search for m/z 522±0.5 Da from the HMDB database resulted in three possible metabolites that were compared for their MS/MS spectra and RT from their standards in Figure S2a. (C) Only lysoPC(18∶1) matched the MS/MS spectra and RT of the plasma spectra (Figure S2a), making it possible to identify the peak at RT 17.2 min and m/z 522.2 as lysoPC(18∶1). (TIF) [file pone.0060556.s002.tif]

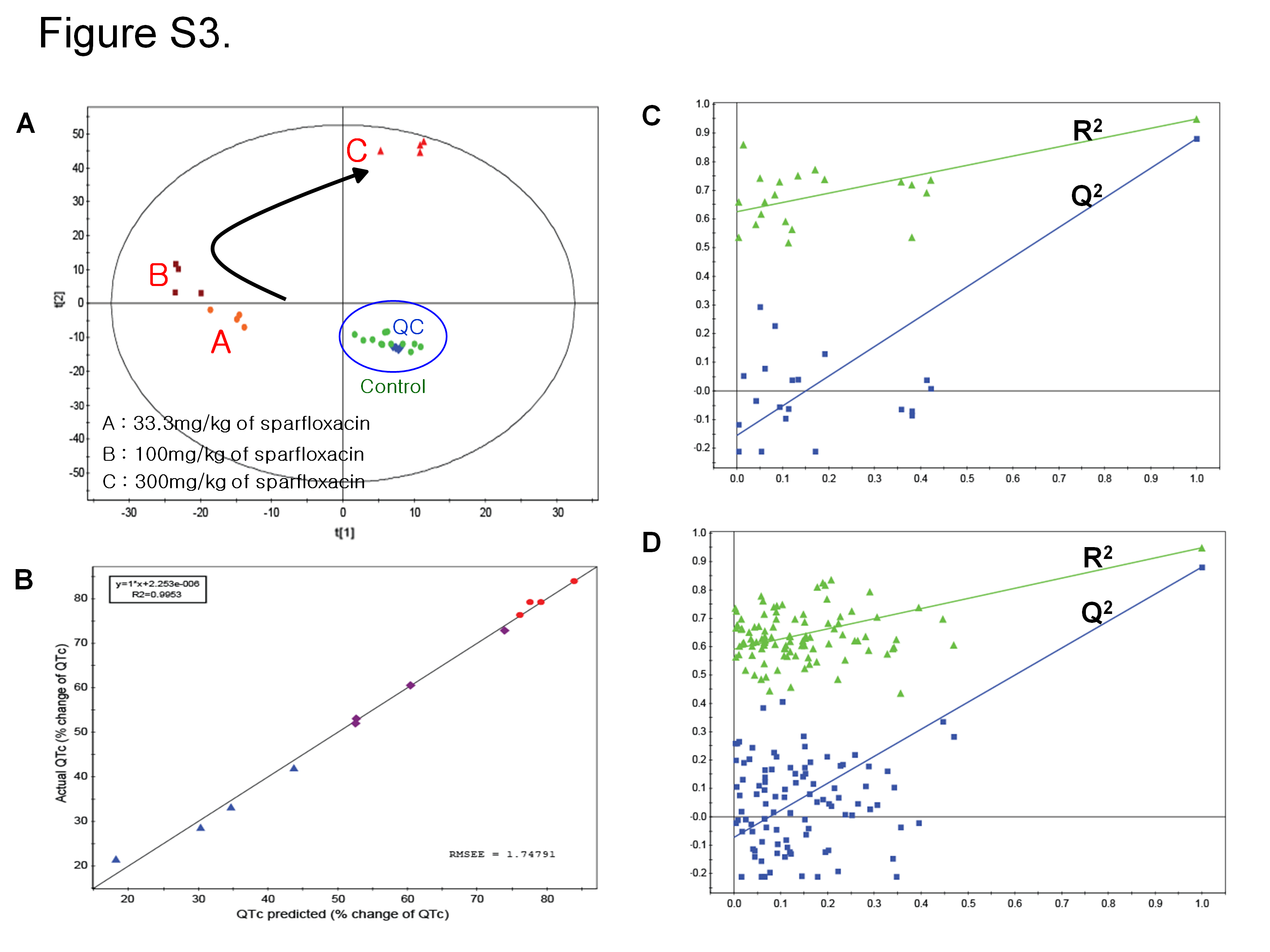

Supplement: Figure S3 — PCA and PLS model validity for predicting drug-induced QT prolongation of sparfloxacin using 12 guinea pig plasma samples (four guinea pigs in each of the low, medium, and high QTc groups). (A) PCA score plot (t[1] vs. t[2]) obtained using the 12 guinea pig samples. We confirmed that the three groups were separated from each other, in the same pattern as in Figure 3A. Eigenvalues were 3.69 and 9.59. Each eigenvalue reflects the dispersion of the corresponding major component. The explanatory powers of t[1] and t[2] were 27.79% and 72.21%, respectively. (B) Plot of predicted QTc versus actual (measured) QTc from the PLS model using a cross-validation method. Predicted values from the PLS model in which all predictions of QTc values showed a linear relationship with actual (measured) QTc values (R2 = 0.9953). (C) Internal validation of the PLS model by 20 permutation tests to confirm predictability and data overfitting showed that all R2 (goodness of fit) and Q2 (predictability of model) values from the permuted models (left) were smaller than those of the original model (far right), demonstrating the validity of the PLS model. (D) Internal validation of the PLS model with 100 permutation tests using stricter validation criteria. (TIF) [file pone.0060556.s003.tif]

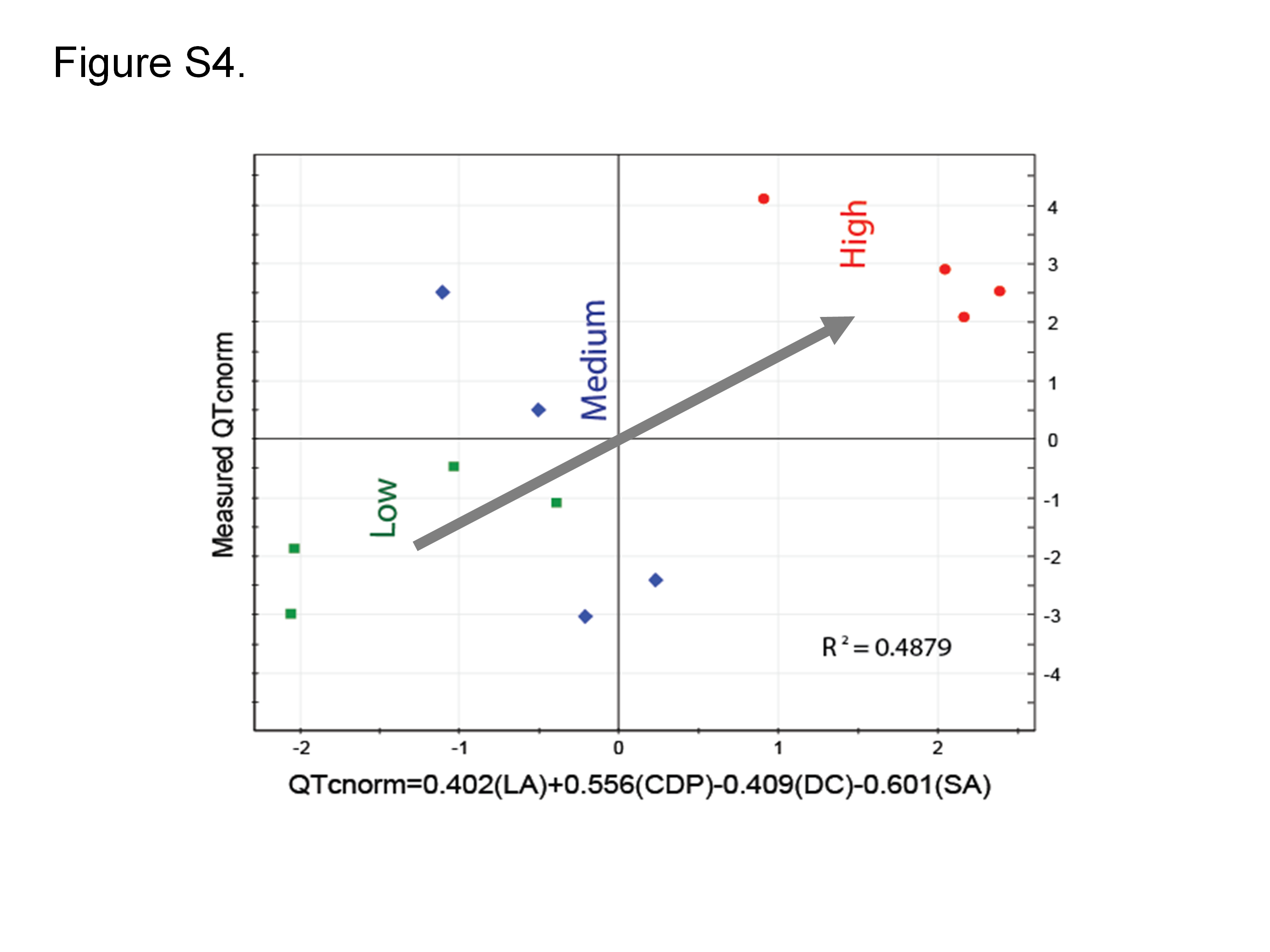

Supplement: Figure S4 — Scatterplot of the predicted normalized QTc values (QTcnorm) from the equation QTcnorm = 0.402(LA)+0.556(CDP)−0.409(DC)−0.601(SA), versus the measured (QTcnorm) values for the 12 samples (four guinea pigs in each of the low, medium, and high groups). Using this prediction index with only four metabolite abundance values, subjects can be categorized into low, medium, and high QTc groups. (TIF) [file pone.0060556.s004.tif]
