# Supplementary material for: Pharmacometabolomic Approach to Predict QT Prolongation in Guinea Pigs
Source: PLoS One. 2013 Apr 4;8(4):e60556. doi: 10.1371/journal.pone.0060556 (PMC3617128; doi:10.1371/journal.pone.0060556)
Supplement: Table S1 — Measured QTcnorm a values (95% CI), and predicted normalized QTc values (95% CI) from the QTcnorm b and QTcnorm c equations for the three guinea pigs not included in the modeling. (DOCX) [file pone.0060556.s005.docx]

**Table S1**

Measured QTc_norm_^a^ values (95% CI), and predicted normalized QTc values (95% CI) from the QTc_norm_^b^ and QTc_norm_^c^ equations for the three guinea pigs not included in the modeling.

| **ID** | **Actual measured QTc (%)** | **Measured QTc_norm_^a^**  **(95% CI)** | **Predicted normalized QTc values (QTc_norm_^b^) from the equation QTc_norm_^b^=0.402(LA)+0.556(CDP)-0.409(DC)-0.601(SA) for the 12 samples**  **(95% CI)** | **Predicted normalized QTc values (QTc_norm_^c^) from the equation QTc_norm_^c^=0.537(LA)+0.533(CDP)-0.431(DC)-0.640(SA) for the 15 samples**  **(95% CI)** |
| --- | --- | --- | --- | --- |
| L1 | 28.6 | -2.73  (-2.38, -0.63) | -2.26  (-2.66, -1.12) | -1.75  (-2.20, -0.55) |
| M1 | 52 | -0.42  (-3.10, 1.27) | -1.30  (-1.68, -0.38) | -0.71  (-1.09, 0.20) |
| H1 | 84 | 2.75  (1.81, 3.03) | 1.63  (0.84, 2.22) | 2.28  (1.50, 2.92) |

CI, confidence interval.
