# Supplementary material for: Pharmacometabolomic Approach to Predict QT Prolongation in Guinea Pigs
Source: PLoS One. 2013 Apr 4;8(4):e60556. doi: 10.1371/journal.pone.0060556 (PMC3617128; doi:10.1371/journal.pone.0060556)
Supplement: Table S2 — Values of the measured QTc (%) calculated by converting the predicted normalized QTc values from the QTcnorm b and QTcnorm c equations for the three guinea pigs not included in the modeling. (DOCX) [file pone.0060556.s006.docx]

**Table S2**

Values of the measured QTc (%) calculated by converting the predicted normalized QTc values from the QTc_norm_^b^ and QTc_norm_^c^ equations for the three guinea pigs not included in the modeling.

| **ID** | **Actual (measured) QTc (%)** | **Measured QTc_norm_^a^** | **Predicted normalized QTc values (QTc_norm_^b^) from the equation QTc_norm_^b^=0.402(LA)+0.556(CDP)-0.409(DC)-0.601(SA) for the 12 samples** | **Predicted normalized QTc values (QTc_norm_^c^) from the equation QTc_norm_^c^=0.537(LA)+0.533(CDP)-0.431(DC)-0.640(SA) for the 15 samples** |
| --- | --- | --- | --- | --- |
| L1 | 28.6 | 28.7 | 29.6 | 35.6 |
| M1 | 52.0 | 52.0 | 43.1 | 49.1 |
| H1 | 84.0 | 84.0 | 75.4 | 80.6 |
